# Supplementary material for: HyperTraPS-CT: Inference and prediction for accumulation pathways with flexible data and model structures
Source: PLoS Comput Biol. 2024 Sep 4;20(9):e1012393. doi: 10.1371/journal.pcbi.1012393 (PMC11404842; doi:10.1371/journal.pcbi.1012393)
Supplement: S1 Appendix — (PDF) [file pcbi.1012393.s001.pdf]

HyperTraPS-CT: Inference and prediction for accumulation pathways with flexible data and model structures

S1 Appendix

Supplementary Information

HyperTraPS in continuous time

To extend HyperTraPS to analyse continuous timing, we first consider the targets of inference and structure of data that we will be dealing with. The state space H of the system is of size 2^L and each state s is labelled by a unique binary string of length L. Let λ be a transition matrix where $\lambda_{i\to j} \equiv \lambda_{ij}$ is the intensity of a transition from state i to state j. All transitions are assumed to be Poissonian. Define a path c in H as a sequence of n states $c_1, ..., c_n$.

If we work in a picture of feature accumulation, dynamics can be envisaged as originating from an 'ancestral' state possessing no features ($000... \equiv 0^L$). Then $\lambda_{i\to j}$ is nonzero only for pairs where j differs from i by exactly one $0 \to 1$ change. In an alternative picture of feature losses, the ancestral state possesses all features ($111... \equiv 1^L$) and $\lambda_{i\to j}$ is nonzero only for pairs where j differs from i by exactly one $1 \to 0$ change. The two pictures can be naturally linked by considering a loss as an 'acquisition of absence'.

To build a framework where both the original and continuous-time cases can be analysed together, we consider as the fundamental quantity of interest $P(b, \tau_1, \tau_2|a, 0; \lambda)$ – *the probability that, having started at state a at time 0, the system will be in state b at a time between τ_1 and τ_2, given a transition matrix λ*. When $\tau_1 = 0, \tau_2 = \infty$, this gives the original HyperTraPS expression for $P(a \to b)$; in the limit $\tau_2 \to \tau_1$, this requires that the system is in state b at the particular time τ_1. From here on, for clarity, we will treat the λ dependence as implicit.

HyperTraPS gives the probability that, having started at a, the system will encounter b (hence, $P(b, 0, \infty|a, 0)$). To compute $P(b, \tau_1, \tau_2|a, 0)$ in more general cases we need additional probabilities, specifically, (i) the probability that we arrive at b before or at τ_1, and do not then move from b until at least τ_1, and (ii) the probability that we arrive at b between τ_1 and τ_2:

$$P(b, \tau_1, \tau_2|a, 0) = \int_{\tau'=0}^{\tau_1} d\tau' P_{arrive}(b, \tau'|a, 0) P_{dwell}(b, \tau_1 - \tau') + \int_{\tau'=\tau_1}^{\tau_2} d\tau' P_{arrive}(\tau'), \quad (1)$$

where P_{arrive} and P_{dwell} respectively give arrival and 'dwell time' probabilities: that is, the probability that b is first reached at time τ', then remains there for at least a further $\tau_1 - \tau'$ period.

It will be useful to define the characteristic 'escape' rate β_i for leaving a point i in the transition network, which is simply the sum of rates of processes leaving that point, hence

$$\beta_i = \sum_s \lambda_{i\to s}. \quad (2)$$

We first consider $P_{dwell}(b, \tau)$, the probability that we dwell at b for at least time τ, which is straightforward to compute. As all the processes by which we can leave b are Poissonian and independent, the process 'leave b by any method' is also Poissonian, with rate β_b. Then the dwell time is exponentially distributed:

Method	Model timing and dependencies	Structure of influences between features	**Input data** (approximate maximum L)	References
OncoTree	Untimed, deterministic	Single events	Cross-sectional (>2000)	[1, 2]
OncoBN	Untimed, deterministic	Multiple AND or OR logic	Cross-sectional (>100)	[3]
CBN	Continuous time, deterministic	Multiple AND logic	Cross-sectional (15); cross-sectional (1000) with H/MC-CBN)	[4, 5]
H-ESCBN (PMCE)	Continuous time, deterministic	Multiple AND, OR, XOR logic	Cross-sectional (14)	[6]
PLI	Discrete time, stochastic	Arbitrary setwise rate influences	Cross-sectional (14)	[7]
HyperTraPS	Discrete time, stochastic	Pairwise rate influences	Cross-sectional, longitudinal, phylogenetic (>120)	[8, 9]
(Tree)MHN	Continuous time, stochastic	Pairwise rate influences	Cross-sectional (25); phylogenetic (>50) with TreeMHN)	[10, 11]
HyperHMM	Discrete time, stochastic	Arbitrary setwise rate influences	Cross-sectional, longitudinal, phylogenetic (25)	[12]

Table A: **Structure of some existing approaches for accumulation modelling.** Further details and an expanded version are given in [13].

$$
\begin{aligned}
P_{dwell}(b,\tau) &= 1 - \int_{\tau'=0}^{\tau} d\tau' P_{leave}(\tau', b; \lambda) && (3)\\
&= 1 - \int_{\tau'=0}^{\tau} d\tau' \beta_b e^{-\beta_b \tau'} && (4)\\
&= e^{-\beta_b \tau}. && (5)
\end{aligned}
$$

We now turn to $P_{arrival}$. Consider a particular path c across the transition network, where steps are Poisson processes with rates $\lambda_{c_i \to c_{i+1}}$, the first state $c_1 \equiv a$, and the nth step reaches a target $c_n \equiv b$. The probability distribution of arrival times at b is then the distribution of the sum of n independent exponentially-distributed random variables describing the waiting times for each step, which is a hypoexponential distribution [14]. For a general set of states:

$$
P(t|\{\beta\}) = \left(\prod_{i=1}^{n} \beta_i \right) \sum_{i=1}^{n} \frac{\exp(-\beta_i t)}{\prod_{j=1, j\neq i}^{n} (\beta_j - \beta_i)}. \quad (6)
$$

Hence, the probability that a path c reaches its final point c_n at time t is

$$
P_{arrival}(t|c) = \left(\prod_{i=1}^{n-1} \beta_{c_i} \right) \sum_{i=1}^{n-1} \frac{\exp(-\beta_{c_i} t)}{\prod_{j=1, j\neq i}^{n-1} (\beta_{c_j} - \beta_{c_i})}. \quad (7)
$$

For clarity we define

$$
\gamma_{c_i} \equiv \prod_{j=1, j\neq i}^{n-1} (\beta_{c_j} - \beta_{c_i}). \quad (8)
$$

It will be useful later to consider

$$
\int_{t'=t_1}^{t_2} dt' P_{arrival}(t'|c) = \left(\prod_{i=1}^{n-1} \beta_{c_i} \right) \sum_{i=1}^{n-1} \left(\frac{1}{\gamma_{c_i} \beta_{c_i}} \left(e^{-\beta_{c_i} t_1} - e^{-\beta_{c_i} t_2} \right) \right) \quad (9)
$$

It is straightforward to verify that setting $t_1 = 0, t_2 = \infty$ gives $\prod_{i=1}^{n-1} \beta_{c_i} \sum_{i=1}^{n-1} \frac{1}{\gamma_{c_i} \beta_{c_i}} = 1$; thus, the probability that the final step is reached at some finite time is 1 unless any $\beta_{c_i} = 0$.

The overall arrival time distribution at b is then given by a weighted sum of this distribution for each path that does indeed arrive at b, hence

$$P_{arrive}(b,t|a,0;\lambda) = \sum_{paths\ c} P(b\ via\ c|a) P_{arrival}(t|c), \tag{10}$$

where $P(b\ via\ c|a)$ is to be determined, but is clearly zero if path c does not lead from a to b.

The generally large number of possible paths on the transition network that do not lead from a to b will lead to this expression being hard to sample naively. Following HyperTraPS, we therefore consider how to make progress sampling only those paths of interest.

Define a state s to be compatible with a state b if, for a picture of evolutionary losses, there exists no i for which $s_i = 0$ and $b_i = 1$ (for the alternative picture of evolutionary acquisitions, this inverts to require no i for which $s_i = 1$ and $b_i = 0$). Thus, s is compatible with b iff b can be reached from s on the hypercube digraph. Define by $B(s)$ the set of states accessible by one step from state s that are compatible with b. HyperTraPS gives us the probability of a path that is guaranteed to encounter b as

$$P(c|b) = \prod_{i=1}^{n-1} P(c_i \to c_{i+1}|c_i \to \circ \in B(c_i)) P(c_i \to \circ \in B(c_i)), \tag{11}$$

where $\circ$ denotes 'any element', and a sampling scheme that encounters only paths leading from a to b, with probability

$$P_{sample}(c) = \prod_{i=1}^{n-1} P(c_i \to c_{i+1}|c_i \to \circ \in B(c_i)). \tag{12}$$

Recording $\prod_i P(c_i \to \circ \in B(c_i))$ through a path sampled by HyperTraPS thus gives an estimate for $P(c|b)$ for that path, which is guaranteed to lead from a to b, and thus gives $P(b\ via\ c|a)$. We can then record $P_{arrival}(t|a \to b\ via\ c)$ for HyperTraPS-sampled paths c and weight each according to its $P(c)$, computed with Eqn. 11:

$$P_{arrive}(b,t|a,0) = \left\langle P_{arrival}(t|a \to b\ via\ c) \prod_i P(c_i \to \circ \in B(c_i)) \right\rangle_{HyperTraPS}. \tag{13}$$

Consider replacing $P_{arrival}(t|a \to b\ via\ c)$ by a simple indicator function $I(c)$ reporting whether path c hits b. The quantity reported will then be the probability that b is reached at any time: $\int_{t'} dt' P_{arrive}(b,t'|a,0)$, or simply $P(a \to b)$. All paths sampled by HyperTraPS will pick up a unit coefficient from the indicator function, and the original HyperTraPS expression for pathway probability is recovered.

We are now in a position to construct the expression required in Eqn. 1:

$$P(b,\tau_1,\tau_2|a,0) = \underbrace{\int_{\tau'=0}^{\tau_1} d\tau' P_{arrive}(b,\tau'|a,0) P_{dwell}(b,\tau_1-\tau')}_{I_1} + \underbrace{\int_{\tau'=\tau_1}^{\tau_2} d\tau' P_{arrive}(\tau')}_{I_2}, \tag{14}$$

where the first integral I_1 accounts for the system arriving at b before τ_1 and dwelling there until τ_1, and the second integral I_2 accounts for the system arriving at b between τ_1 and τ_2.

The first integral is given by

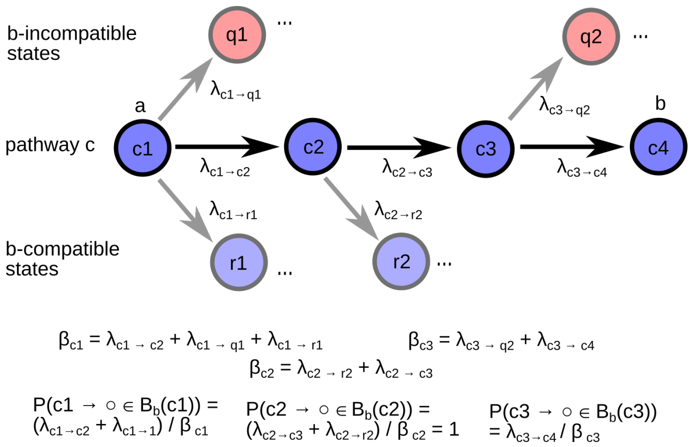

Figure A: **Computing β and α for a pathway c.** An illustrative pathway running from $a \equiv c_1$ to $b \equiv c_4$. At each step, alternative transitions may exist to b-compatible states (r_1, r_2) and/or b-incompatible states (q_1, q_2). The escape rate β_s for a given step on the pathways is the sum of rates leaving that state; the compatibility probability $P(s \to \circ \in B_b(s))$ is the probability of undergoing a transition to a b-compatible state. The compatibility product α is the product of this probability over every step in the pathway. α and the set of β values are the statistics of a pathway that are used in constructing the estimated transition probability (Eqn. 1).

$$
\begin{aligned}
I_1(b, \tau_1|a, 0) &= \int_{\tau'=0}^{\tau_1} d\tau' P_{arrive}(b, \tau'|a, 0) P_{dwell}(b, \tau_1 - \tau') &(15)\\
&= \int_{\tau'=0}^{\tau_1} d\tau' \left(\sum_{all\ c} P(b\ via\ c|a) P_{arrival}(\tau'|a \to b\ via\ c) \right) P_{dwell}(b, \tau_1 - \tau') &(16)\\
&= \int_{\tau'=0}^{\tau_1} d\tau' \left(\sum_{c\ from\ a \to b} P(c) P_{arrival}(\tau'|a \to b\ via\ c) \right) P_{dwell}(b, \tau_1 - \tau') &(17)\\
&= \int_{\tau'=0}^{\tau_1} d\tau' \left(\sum_{c\ from\ a \to b} \left(\prod_i^{n-1} \frac{\lambda_{c_i \to c_{i+1}}}{\beta_{c_i}} \right) \left(\prod_{i=1}^{n-1} \beta_{c_i} \right) \left(\sum_{i=1}^{n-1} \frac{\exp(-\beta_{c_i}\tau')}{\prod_{j=1, j\neq i}^{n-1} (\beta_{c_j} - \beta_{c_i})} \right) \right) e^{-\beta_b(\tau_1 - \tau')} &(18)\\
&\simeq \int_{\tau'=0}^{\tau_1} d\tau' \left\langle \left(\prod_i P(c_i \to \circ \in B(c_i)) \right) \left(\prod_{i=1}^{n-1} \beta_{c_i} \right) \sum_{i=1}^{n-1} \frac{\exp(-\beta_{c_i}\tau')}{\prod_{j=1, j\neq i}^{n-1} (\beta_{c_j} - \beta_{c_i})} \right\rangle_C e^{-\beta_b(\tau_1 - \tau')} &(19)
\end{aligned}
$$

where the angle brackets denote sampling from a set of paths C using HyperTraPS. Noting that $\langle f(c) \rangle_C = f(c)N(c)/N_h$, where $N(c)$ is the number of occurrences of path c in C and N_h is the total number of HyperTraPS samples in C, this integral can be evaluated to give an estimate for $P(b, \tau_1, \tau_2|a, 0)$:

$$
\begin{aligned}
\hat{I}_1(b, \tau_1|a, 0) &= \int_{\tau'=0}^{\tau_1} d\tau' e^{-\beta_b \tau_1} e^{\beta_b \tau'} \sum_c \frac{N(c)}{N_h} \alpha(c) \sum_i v_i(c) e^{-\beta_{c_i}\tau'} &(20)\\
&= e^{-\beta_b \tau_1} \sum_c \frac{N(c)}{N_h} \alpha(c) \sum_i \left(\frac{v_i(c)(1 - e^{-\tau_1(\beta_{c_i} - \beta_b)})}{\beta_{c_i} - \beta_b} \right), &(21)
\end{aligned}
$$

where

$$\alpha(c) \equiv \prod_{i=1}^{n-1} P(c_i \to \circ \in B(c_i)); \quad (22)$$

$$v_i(c) \equiv \left(\prod_{j=1}^{n-1} \beta_{c_j} \right) \frac{1}{\prod_{j=1, j\neq i}^{n-1} (\beta_{c_j} - \beta_{c_i})} \quad (23)$$

for c, a particular path sampled by HyperTraPS, and where the required α and β_{c_i} values can be easily recorded during a sampling algorithm.

If $a \equiv b$, the situation reduces to the case where we have arrived at b at $t = 0$, equivalent to $P_{arrive}(b, \tau'|a, 0) = \delta(\tau')$ in Eqn. 15. We are then only concerned with dwelling at b for the remaining time t:

$$P(b, \tau_1|b, 0) = P_{dwell}(b, \tau_1) = \exp(-\beta_b \tau_1) \quad (24)$$

The second integral, I_2, is given by

$$I_2(b, \tau_1, \tau_2|a, 0) = \int_{\tau'=\tau_1}^{\tau_2} d\tau' P_{arrive}(\tau') \quad (25)$$

$$\simeq \int_{\tau'=\tau_1}^{\tau_2} d\tau' \left\langle \left(\prod_i P(c_i \to \circ \in B(c_i)) \right) \left(\prod_{i=1}^{n-1} \beta_{c_i} \right) \sum_{i=1}^{n-1} \frac{\exp(-\beta_{c_i}\tau')}{\prod_{j=1, j\neq i}^{n-1} (\beta_{c_j} - \beta_{c_i})} \right\rangle_C, \quad (26)$$

by analogy with the above derivation. Using Eqn. 9, this integral can also be solved to give

$$\hat{I_2}(b, \tau_1, \tau_2|a, 0) = \int_{\tau'=\tau_1}^{\tau_2} d\tau' \sum_c \frac{N(c)}{N_h} \alpha(c) P_{arrival}(\tau'|c) \quad (27)$$

$$= \sum_c \frac{N(c)}{N_h} \alpha(c) \left(\prod_{i=1}^{n-1} \beta_{c_i} \right) \sum_{i=1}^{n-1} \left(\frac{1}{\gamma_{c_i} \beta_{c_i}} \left(e^{-\beta_{c_i}\tau_1} - e^{-\beta_{c_i}\tau_2} \right) \right) \quad (28)$$

$$= \sum_c \frac{N(c)}{N_h} \alpha(c) \sum_i \left(\frac{v_i(c)}{\beta_{c_i}} \left(e^{-\beta_{c_i}\tau_1} - e^{-\beta_{c_i}\tau_2} \right) \right), \quad (29)$$

where, as before, the required α and β_{c_i} values can be easily recorded during a sampling algorithm.

Together we finally obtain

$$\hat{P}(b, \tau_1, \tau_2|a, 0)$$

$$= I_1 + I_2 \quad (30)$$

$$= \sum_{c\in C} \frac{N(c)}{N_h} \alpha(c|b) \left(\left(e^{-\beta_b\tau_1} \sum_i \left(\frac{v_i(c)(1 - e^{-\tau_1(\beta_{c_i} - \beta_b)})}{\beta_{c_i} - \beta_b} \right) \right) + \sum_i \left(\frac{v_i(c)}{\beta_{c_i}} \left(e^{-\beta_{c_i}\tau_1} - e^{-\beta_{c_i}\tau_2} \right) \right) \right). \quad (31)$$

If complete sampling of the set of paths from a to b is possible, we can obtain an exact expression for $P(b \; via \; c|a)$ in Eqn. 10. This probability is simply $P(c)I(c|a, b)$, where $P(c) = \prod_i P(c_i \to c_{i+1})$ and $I(c|a, b)$ returns 1 if path c starts at a and ends at b and 0 otherwise. Following Eqn. 10, this expression can be substituted in Eqn. 23 for the approximate sampling weight $N(c)/N_h$, giving

$$P(b, \tau_1, \tau_2|a, 0) = \sum_c P(c)I(c|b, a) \sum_i \left(e^{-\beta_b\tau_1} \frac{v_i(c)(1 - e^{-\tau_1(\beta_{c_i} - \beta_b)})}{\beta_{c_i} - \beta_b} + \frac{v_i(c)}{\beta_{c_i}} \left(e^{-\beta_{c_i}\tau_1} - e^{-\beta_{c_i}\tau_2} \right) \right) \quad (32)$$

We can then run the HyperTraPS sampling algorithm to perform inference (i) in the absence of temporal information ($\tau_1 = 0, \tau_2 = \infty, I_1 = 0, I_2 = P(a \to b)$); (ii) for precisely defined temporal samples ($\tau_1 = \tau_2 = \tau, I_1 = P(b, \tau|a, 0), I_2 = 0$); and (iii) for uncertain temporal samples ($0 \leq \tau_1 < \tau_2 < \infty, I_1, I_2$ nonzero). Case (i) corresponds to original HyperTraPS. We note that the posterior orderings obtained from (i), (ii), and (iii) may in general differ.

Inference using HyperTraPS-CT likelihood estimation

Bayesian or maximum likelihood inference

Previous implementations of (discrete-ordering) HyperTraPS used a Bayesian approach with MCMC to identify transition matrices (given uniformative prior distributions) compatible with observations [9, 8, 7]. This process can naturally be extended to the continuous time picture (Fig. 1C).

Algorithm 2. Inference of posterior transition matrices using HyperTraPS-CT. Requires data $\mathcal{D}$, prior distribution $P_{prior}(\lambda)$, MCMC parameters θ.

1. Choose an initial parameterisation λ (for example, all permitted $\lambda_{i \to j}$ equal).
2. Iterate:
   (a) Select $\lambda' = \lambda + \Delta$, where Δ is a random perturbation specified by θ.
   (b) Compute $h = \min\left(1, \frac{\mathcal{L}(\mathcal{D}|\lambda')P_{prior}(\lambda')q(\lambda' \to \lambda)}{\mathcal{L}(\mathcal{D}|\lambda)P_{prior}(\lambda)q(\lambda \to \lambda')}\right)$, where q is a transition kernel specified by θ, and $\mathcal{L}$ is computed using Eqn. 31 and Algorithm 1.
   (c) Set $\lambda \to \lambda'$ with probability h.
   (d) If conditions for sampling described in θ are met, record λ as a sample from $P_{post}(\lambda)$.
3. end iterate.

We note that in practise it is usually more convenient to work with log-likelihoods $\ell = \log \mathcal{L}$ than with $\mathcal{L}$. We typically use uninformative uniform priors over $\log \lambda_{ij}$, and allow the parameters of the process above to be set by the user to best support converged inference results for a given dataset. A rule-of-thumb first choice is a perturbation kernel $\mathcal{N}(0, 0.05)$ for $\log \lambda_{ij}$, $N_h = 200$ and 10^4 MCMC iterations (discarding the first 20% as burn-in).

If a maximum likelihood perspective is more desirable for reasons of computational time and/or interpretation, the likelihood estimate from HyperTraPS-CT can readily be maximised over parameters using any numerical optimiser. We include simulated annealing and a variant of stochastic gradient descent in the HyperTraPS-CT code; the behaviour of these approaches for the double-pathway test case is shown in Fig. C.

Parameter spaces

As L increases, the dimensionality of λ increases considerably. For tractability and/or interpretation, it may desirable to impose relationships between the different edges on the hypercubic transition network, and so decrease the dimensionality of the parameter space involved.

We begin by considering the dimensionality-reduction approach in [15] and [16], independently published as mutual hazard networks [10]. Here, an $L \times L$ matrix is used to encode intensities for individual feature losses and the influence of one feature's presence or absence on the loss intensity of another. Higher-order effects are neglected, and we no longer have an independent and unconstrained $\lambda_{i \to j}$ intensity for each transition, but the scaling of parameter space size with L is reduced from exponential to polynomial and subtle dynamics can still be captured (see below and [8]). Specifically, if $s^{(i)}$ is the binary absence/presence value of the ith trait in state s,

$$\lambda_{s_i \to s_j} = \begin{cases} \exp\left(\pi_{kk} + \sum_{l \neq k} s_i^{(l)} \pi_{kl}\right) & \text{if } s_i \text{ and } s_j \text{ differ only at locus } k \text{ and } s_i^{(k)} = 0; \\ 0 & \text{otherwise.} \end{cases} \quad (33)$$

Hence, the diagonal elements π_{kk} encode a basal rate with which trait k is acquired, and the off-diagonal elements π_{kl} encode how the presence of the lth trait influences (positively or negatively) the rate with which trait k is acquired.

For example, $\pi_{11} = 0, \pi_{ij} = \Delta$ if $j - 1 = 1$, $\pi_{ij} = -\Delta$ otherwise, encodes a transition matrix with strong support for a single pathway, where trait 1 is acquired first (rate $\pi_{11} = e^0$ against $e^{-\Delta}$ for other traits). The rate of acquisition of trait 2 then increases to $\pi_{22} + \pi_{12} = 0$, making trait 2 the next most likely acquisition, and so on.

For the next case, we consider a 3rd-order array π_{ijk}. Then the 'base rate' for k is π_{kkk}, the influence of j on k is π_{jjk}, and the independent influence of the pair (i, j) on k is $\pi_{ijk} = \pi_{jik}$. Clearly in this, and higher-order, cases, it is not true that every element of the corresponding array is independent: symmetries amongst the definitions of subsets (pair (i, j) is identical to pair (j, i)) mean that some elements have the same meaning. But in general we can use a subset of elements in the nth-order array to encode influences from single features, pairs of features, triples of features, and so on, as well as their 'target' base acquisition rate.

For the limiting case where transitions between states are completely independent – corresponding, in the feature picture, to arbitrary influences of subsets of all sizes – we simply use an array of size $L2^{L-1}$, the cardinality of the edge set of the L-hypercube, to store the individual rates.

Validation, inclusion of prior information, and analysis of posteriors

To test the HyperTraPS-CT algorithm, we first tested the ability of Eqn. 1 and Algorithm 1 to estimate the probabilities underlying accumulation dynamics in continuous time. To this end, we constructed a simple $L = 3$ model system and considered the probability that the system was in state 110 at time $t = \tau$ given that it was in 000 at time $t = 0$, for a set of randomly-chosen parameterisations λ. For this simple system, analytic results are readily available and uniform sampling also yields a good estimate of this probability over time. Fig. BA shows that HyperTraPS-CT (with $N_h = 10^3$) readily matches these results, demonstrating the accuracy of the approach in inferring evolutionary timescales.

We next tested the ability of Algorithm 2 to infer parameters underlying evolutionary observations. We constructed a synthetic dataset from a model system with $L = 3$ and a fixed set of transition rates (Fig. BB). This synthetic system models a case where a slow first transition facilitates a fast second transition, and then a final transition occurs more slowly. We simulated $n = 10^3$ independent observations at $t_1 \sim \mathcal{U}(0, 20), t_2 \sim \tau_1 + \mathcal{U}(0, 10)$ from this system and used these synthetic observations with Algorithm 2 to produce posterior distributions on the underlying parameters. Fig. BB shows the results of this process, where HyperTraPS-CT readily recovers the dynamic structure of the evolutionary system. We also simulated $n = 10^3$ independent synthetic observations from an $L = 3$ with a full set of randomly chosen transition rates and $t_1 \sim \mathcal{U}(0, 2), t_2 \sim t_1 + \mathcal{U}(0, 5)$. HyperTraPS-CT recovers the underlying parameters reliably, with few posteriors exhibiting a mode substantially differing from the true value (Figs. BC, D).

This validation experiment serves to emphasise a principle in the analysis of posteriors produced by HyperTraPS. In some contexts, some processes in the transition network may be very weakly constrained by data – for example, the $010 \to 110$ and $011 \to 111$ transitions in Fig. BB (labelled $2 \to 6$ and $3 \to 7$ respectively). In such cases, where systems are highly constrained to follow only a subset of paths, a heuristic can be employed for simplification: set to zero the rates of any processes that are encountered in a proportion lower than α of trajectories. For example, $\alpha = 0.01$ would remove all steps that had a less than 1% probability of occurring. As L increases, the probability of specific pathways may be expected to decrease entropically, and choices for α should be made

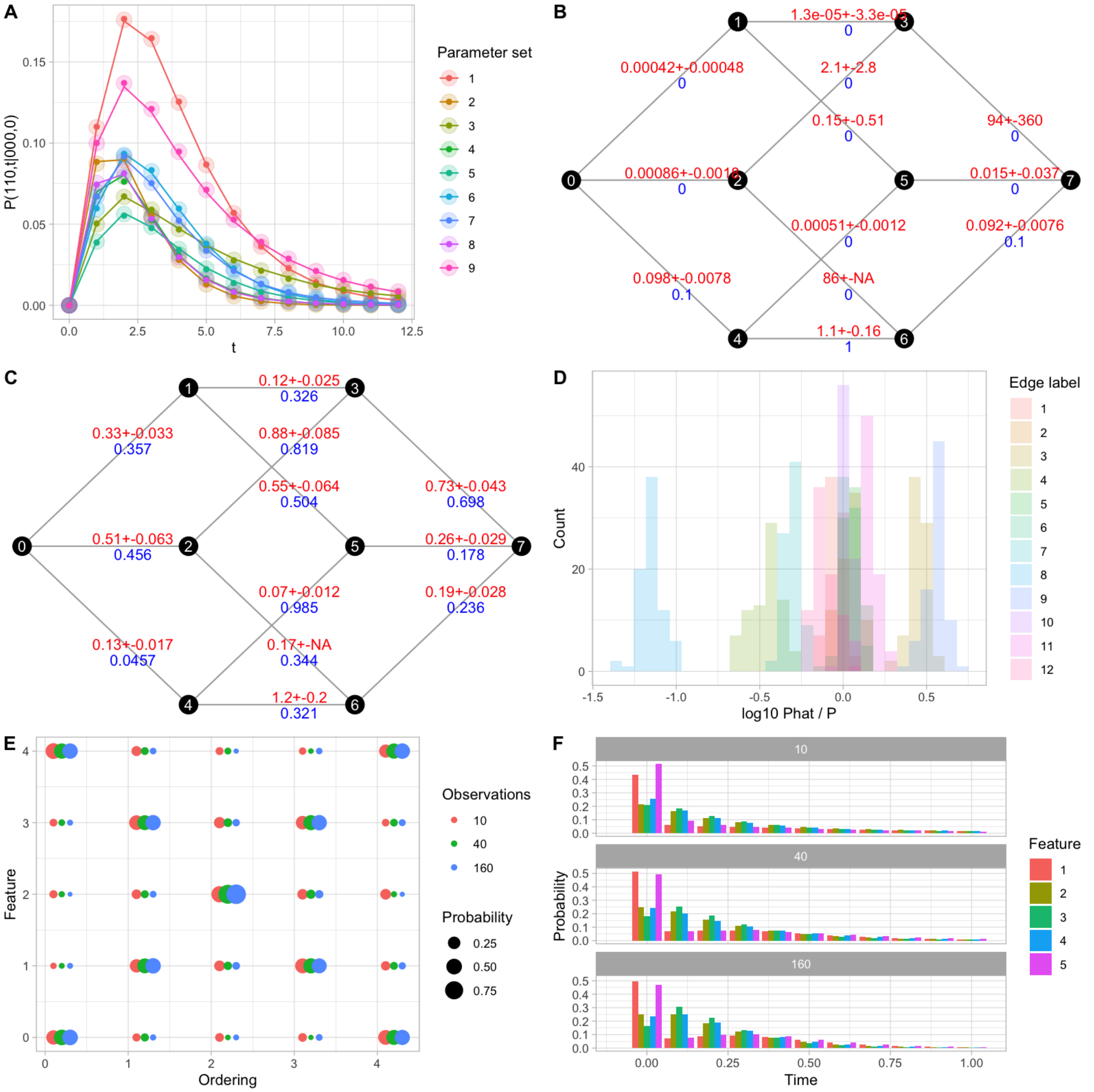

Figure B: **Validation and demonstration of HyperTraPS-CT** (see text in 'Validation, inclusion of prior information, and analysis of posteriors' below). (A) Estimation of transition timescales and probabilities using HyperTraPS-CT (Eqn. 1; Algorithm 1, solid points) matches results from analytic calculation (lines) and exhaustive sampling (transparent points) of a tractable $L = 3$ model system under many different random parameterisations (each trace corresponds to a different parameterisation). (B) HyperTraPS-CT accurately recovers the original transition rate parameters for a given $L = 3$ system. Here, states are represented by their decimal equivalent (0 is 000, 6 is 110). Blue figures give the true system; red shows inferred means and 95% credibility intervals for each transition. (C) HyperTraPS-CT recovers original transition rates for a more complex model system; plots show original and inferred rates as in (B). (D) Inferred distributions of transition weights from (C). Histograms show the posterior distributions on each transition rate $\hat{P}$ normalised by its true value P; most distributions (blue) have modes in a fold range around the true value. (E) *Feature acquisition orderings*: HyperTraPS-CT recovers posterior orderings of events for a model $L = 5$ system constructed to allow two competing paths (corresponding to an X-shaped structure in this plot). Smaller sample sizes ($n = 10$) lead to increased uncertainty in the pathway structures; higher observation numbers readily identify pathways. (F) *Feature acquisition orderings:* Inferred timing distributions of feature acquisitions in the experiments in (E); in the generating process, each step takes 0.1 time units.

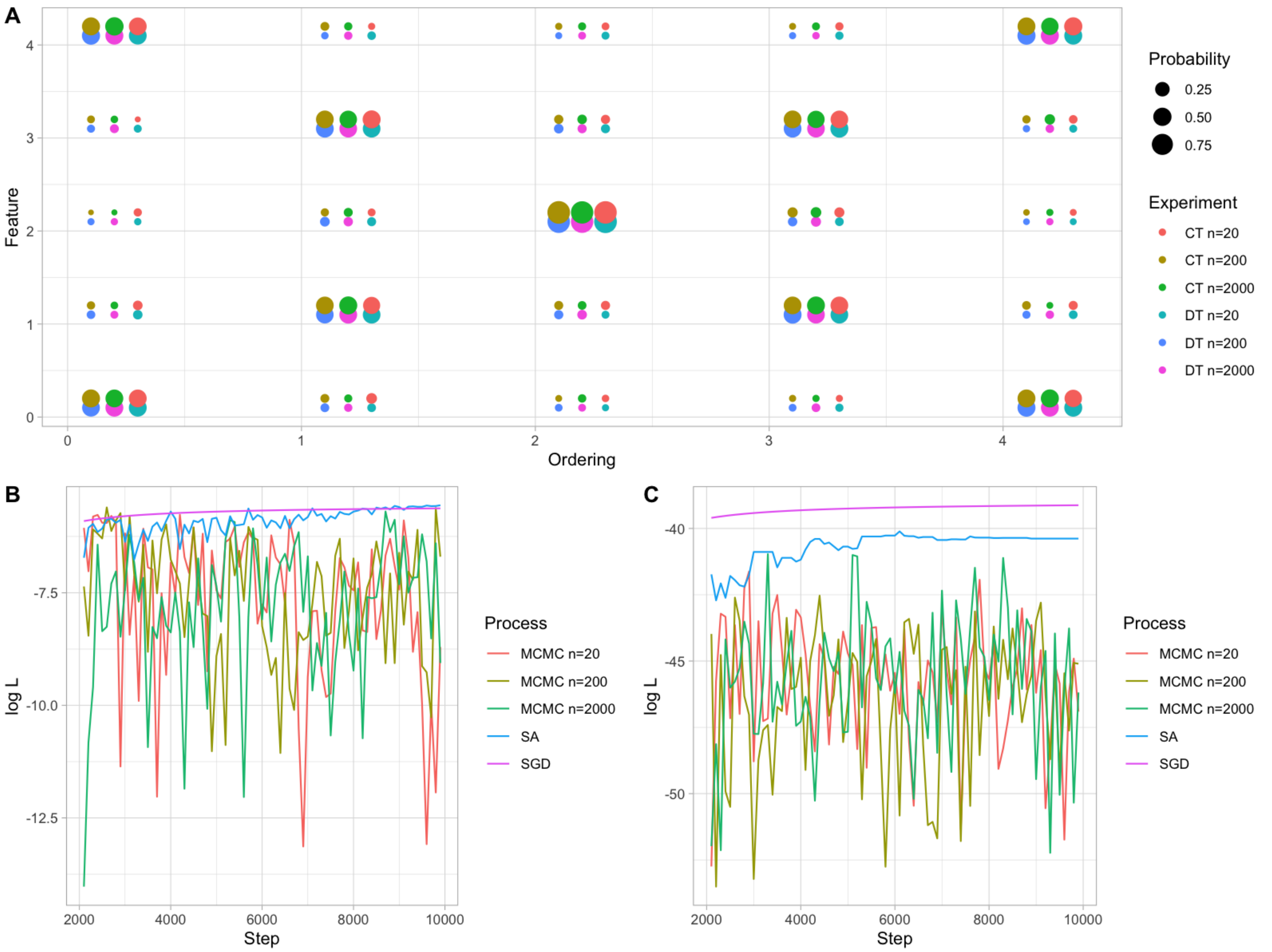

Figure C: **Continuous and discrete time HyperTraPS inference.** Inference performed with different numbers of sampling walkers N_h, for the same two-pathway test model as in Supp. Fig. B. (A) *Feature acquisition orderings* as a 'bubble plot' for the different approaches: CT (continuous time) and DT (discrete time, original HyperTraPS); different walker counts $n = N_h$. (B-C) Likelihood throughout algorithm progress for (B) discrete time (C) continuous time, for MCMC chains with different numbers of sampling walkers, stochastic gradient descent (SGD), and simulated annealing (SA) optimisers.

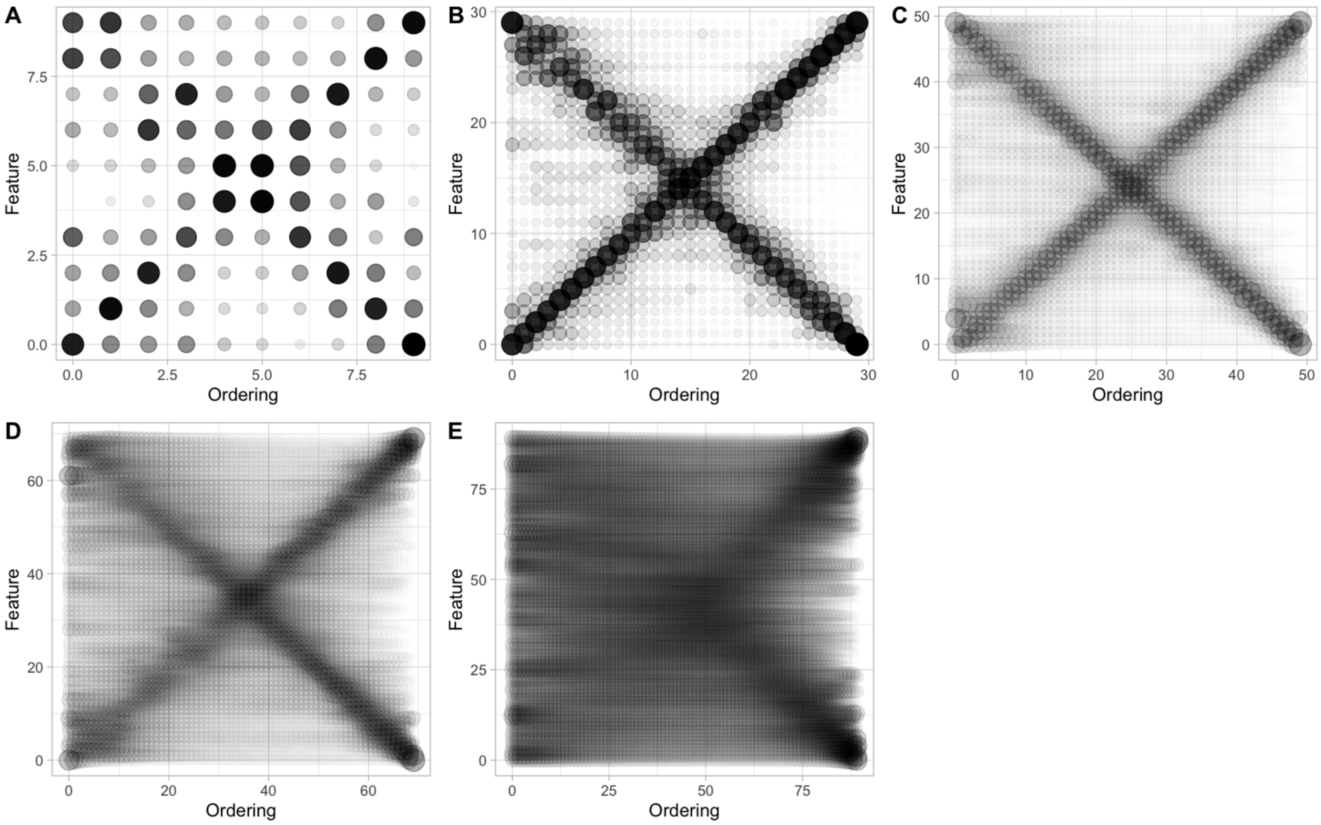

Figure D: **Inference with number of features.** (A-E) *Feature acquisition orderings* showing posteriors on event ordering from discrete time HyperTraPS with increasing numbers of feature $L = 10, 30, 50, 70, 90$ and dataset size $2L$. The size of a point gives the probability that a given feature (vertical axis) is acquired at a given step in the accumulation process (horizontal axis). The two-pathway structure generated the data in each case.

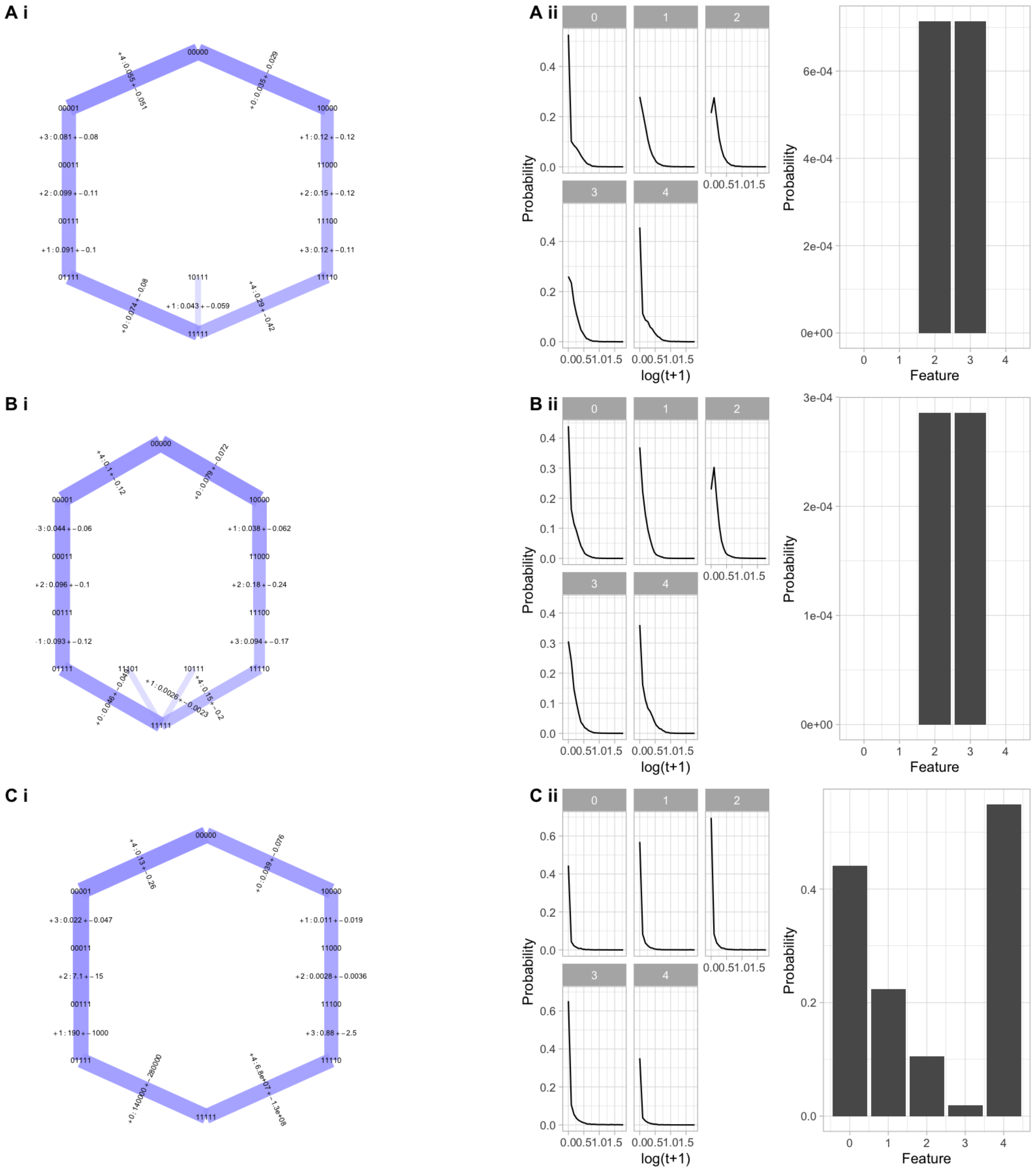

Figure E: **Influence of precision or uncertainty on transition timings.** Here, inference is performed using the same illustrative competing-pathway system as throughout the manuscript, but the distribution of timings of each event are specified from different distributions. If the ‘true’ generating process gave timing τ_i for the ith observation, these were (A) precisely specified, $[\tau_i, \tau_i]$; (B) uncertain time window, $[\tau_i/4, 4\tau_i]$, (C) infinite time window, $[0, \inf]$. (C) corresponds to the case where only orderings, not absolute timings, are considered. (i) *Hypercubic transition networks* with mean and s.d. for the timing of each step; (ii) *feature acquisition orderings* where histograms give the distribution of acquisition times associated with each feature, and bar plots give the probability that acquisition does not occur within a threshold time (here, 3 time units).

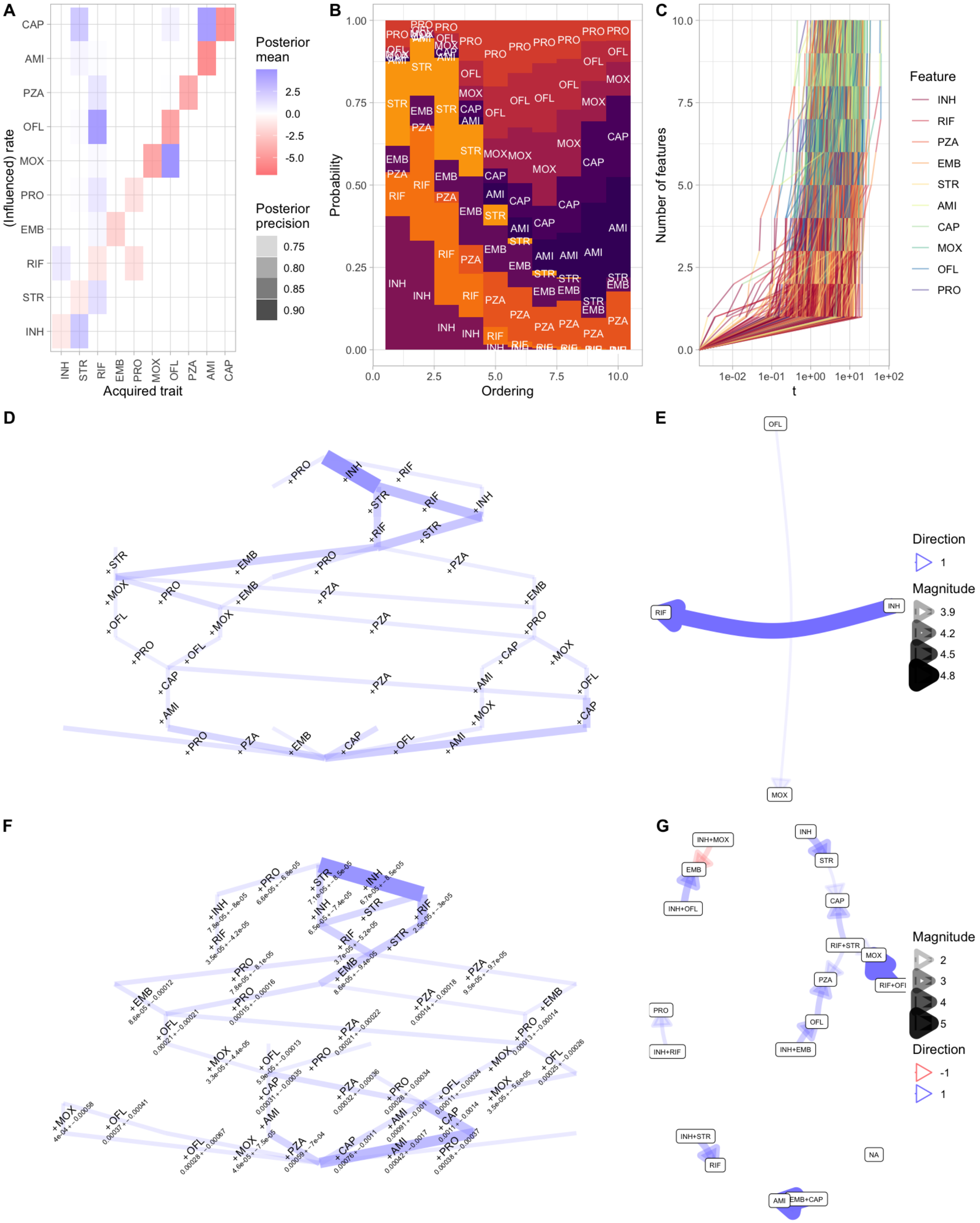

Figure F: **Additional and alternative outputs of inference for the anti-microbial resistance evolution system.** (A-C) Additional plots accompanying Fig. 5: (A) *Influence matrix* map of inferred influences between drug resistances; (B) *Feature acquisition orderings* via motif plot giving the probability that a given feature is acquired at a particular ordering in the accumulation process; (C) *Feature acquisition orderings* via an ensemble of sampled time series of the evolutionary process; here 'time' corresponds to the amount of mutational change in the original phylogenetically-embedded data. (D) *Hypercubic transition network* for the discrete time case, ignoring timing information, mirroring previous findings [9] and with less emphasis on early STR (streptomycin) acquisition. (E-F) Output including uncertainty on the branch lengths in the source data phylogeny: (E) *Influence graph* showing a more limited network of reliably inferred influence; (F) *Hypercubic transition network* showing transition timings with lower modal values. (G) *Influence graph* showing inferred influences of pairs of features in the L^3 system, thresholded by a posterior coefficient of variation of 0.3.

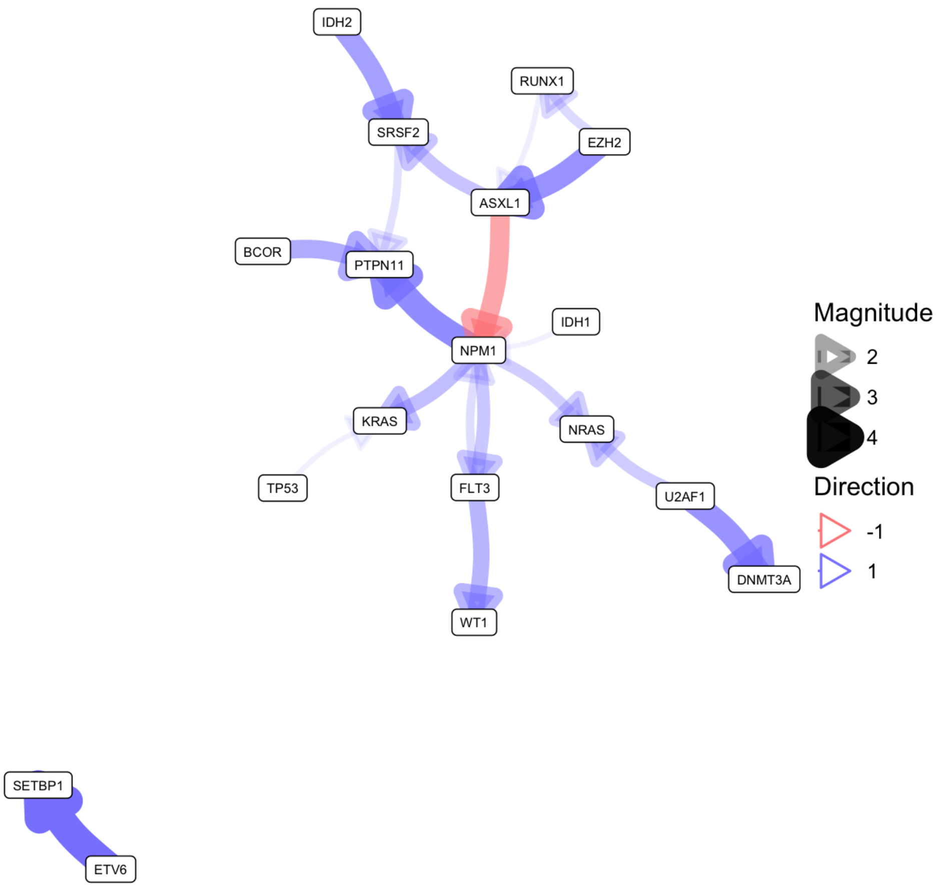

Figure G: ***Influence graph* giving a network visualisation of influences between feature acquisitions in the cancer progression case study.**

appropriately. This 'thresholding' approach has recently been used to construct a distance metric between inferred hypercubic transition networks [17].

We next tested the ability of HyperTraPS-CT to infer distinct evolutionary pathways given observations coupled by a phylogenetic relationship. We considered a larger test case in this instance, by constructing an $L = 5$ systems supporting two competing pathways 'right-first' $00000 \to 00001 \to 00011...$, and 'left-first' $00000 \to 10000 \to 11000....$ We constructed synthetic datasets of varying size by simulating outputs from this network. Observations were recorded as ancestor-descendant end states, modelling the phylogenetic reconstruction we use throughout this article and in [8].

Figs. BE-F show the ability of HyperTraPS-CT to reproduce these constrained pathways. The resulting posterior ordering distributions (Fig. BE) show that HyperTraPS-CT recovers the double pathway structure perfectly for large sets of observations, and very satisfactorily for smaller sets. Fig. BF shows histograms of the inferred continuous timing of each feature acquisition, well recovering the original timing of 0.1 time units per event.

Fig. C shows the behaviour of HyperTraPS and HyperTraPS-CT with different trajectory sample counts N_h and used within different schemes for parameter inference: MCMC for a Bayesian approach, and maximum likelihood via simulated annealing and stochastic gradient descent. Fig. D shows the behaviour as the number of features L in the dataset is increased; for each L, $2L$ observations are produced, so each transition on the hypercube has exactly one associated observation. Even for this limited observation set, the structure of the underlying network is well captured for large feature sets L.

References

[1] Richard Desper, Feng Jiang, Olli-P Kallioniemi, Holger Moch, Christos H Papadimitriou, and Alejandro A Schäffer. Inferring tree models for oncogenesis from comparative genome hybridization data. *Journal of computational biology*, 6(1):37–51, 1999.

[2] Aniko Szabo and Kenneth Boucher. Estimating an oncogenetic tree when false negatives and positives are present. *Mathematical biosciences*, 176(2):219–236, 2002.

[3] Phillip B Nicol, Kevin R Coombes, Courtney Deaver, Oksana Chkrebtii, Subhadeep Paul, Amanda E Toland, and Amir Asiaee. Oncogenetic network estimation with disjunctive Bayesian networks. *Computational and Systems Oncology*, 1(2):e1027, 2021.

[4] Moritz Gerstung, Michael Baudis, Holger Moch, and Niko Beerenwinkel. Quantifying cancer progression with conjunctive bayesian networks. *Bioinformatics*, 25(21):2809–2815, 2009.

[5] Hesam Montazeri, Jack Kuipers, Roger Kouyos, Jürg Böni, Sabine Yerly, Thomas Klimkait, Vincent Aubert, Huldrych F Günthard, Niko Beerenwinkel, and Swiss HIV Cohort Study. Large-scale inference of conjunctive Bayesian networks. *Bioinformatics*, 32(17):i727–i735, 2016.

[6] Fabrizio Angaroni, Kevin Chen, Chiara Damiani, Giulio Caravagna, Alex Graudenzi, and Daniele Ramazzotti. PMCE: efficient inference of expressive models of cancer evolution with high prognostic power. *Bioinformatics*, 38(3):754–762, 2022.

[7] B. Williams, I. Johnston, S. Covshoff, and J. Hibberd. Phenotypic landscape inference reveals multiple evolutionary paths to C4 photosynthesis. *Elife*, 2, 2013.

[8] Iain G Johnston and Ben P Williams. Evolutionary inference across eukaryotes identifies specific pressures favoring mitochondrial gene retention. *Cell systems*, 2(2):101–111, 2016.

[9] Sam F Greenbury, Mauricio Barahona, and Iain G Johnston. HyperTraPS: inferring probabilistic patterns of trait acquisition in evolutionary and disease progression pathways. *Cell systems*, 10(1):39–51, 2020.

[10] Rudolf Schill, Stefan Solbrig, Tilo Wettig, and Rainer Spang. Modelling cancer progression using mutual hazard networks. *Bioinformatics*, 36(1):241–249, 2020.

[11] Xiang Ge Luo, Jack Kuipers, and Niko Beerenwinkel. Joint inference of exclusivity patterns and recurrent trajectories from tumor mutation trees. *Nature Communications*, 14(1):3676, 2023.

[12] Marcus T Moen and Iain G Johnston. HyperHMM: efficient inference of evolutionary and progressive dynamics on hypercubic transition graphs. *Bioinformatics*, 39(1):btac803, 2023.

[13] Ramon Diaz-Uriarte and Iain G Johnston. A picture guide to cancer progression and monotonic accumulation models: evolutionary assumptions, plausible interpretations, and alternative uses. *arXiv preprint arXiv:2312.06824*, 2024.

[14] Sheldon M Ross. *Introduction to probability models*. Academic press, 2014.

[15] Iain G Johnston and Nick S Jones. Evolution of cell-to-cell variability in stochastic, controlled, heteroplasmic mtDNA populations. *The American Journal of Human Genetics*, 99(5):1150–1162, 2016.

[16] M. Hjelm, M. Höglund, and J. Lagergren. New probabilistic network models and algorithms for oncogenesis. *Journal Of Computational Biology*, 13:853, 2006.

[17] Belén García Pascual, Lars M Salbu, Jessica Renz, Konstantinos Giannakis, and Iain G Johnston. Comparing structure and dynamics of transition graphs by the symmetric difference metric over an edge-filtration. *IEEE Access*, 12:81030–81046, 2024.
