# Supplementary material for: HyperTraPS-CT: Inference and prediction for accumulation pathways with flexible data and model structures
Source: PLoS Comput Biol. 2024 Sep 4;20(9):e1012393. doi: 10.1371/journal.pcbi.1012393 (PMC11404842; doi:10.1371/journal.pcbi.1012393)
Supplement: Attachment — Submitted filename: hct-reviewers-rev.pdf [file pcbi.1012393.s002.pdf]

We’re very grateful to both reviewers for their positive feedback and useful comments!

Reviewer #1: The paper reports an extension to a sequence of results and tools the authors have worked on in the past years. The *HyperTraPS-CT* tool extends the sampling methods the authors have worked on, by adding the capability to include (continuous) wall time in its core algorithms. The added capabilities allow HyperTraPS-CT to expand its posterior probability constructions to a wide range of phenomena and systems where a temporal model reconstruction is needed. In particular the authors name *cross-sectional*, *phylogenetic* and *longitudinal* data where various combinations of features can be analyzed (combination can be ternary and beyond).

The paper concludes by showing the application of HyperTraPS-CT to two recent and varied datasets, one the well-known Morita's SCs AML dataset, used to infer cancer progression, and a second one to check acquired resistance in tuberculosis. The results reported in the applications do make sense and are useful as supporting arguments about the goodness of the approach.

The paper is very well written and full of well presented information. Each choice presented is properly contextualized and explained. The resulting tool, HyperTraPS-CT appears to be significant improvement over the state of the art, even considering the previous non-CT version. I have not seen paper of this quality and usefulness in a while.

Thanks immensely for this positive feedback, and for your useful and thoughtful points below!

Nevertheless, I do have a few suggestions for improvement.

Although this is implied in much of the paper and of the state of the art, it would be useful to clearly state the overall computational complexity of the algorithms making up HyperTraPS-CT.

We’ve given the explicit scaling of the likelihood estimation (O(n L^2 N_h), where n = number of observations, L = number of features, N_h = number of samplers)), and discussed how this relates to the scaling of the inference process overall.

On a related point, I would suggest the authors to include a separate "box" explaining the different sampling strategies mentioned in the

state of the art. This would make the paper more self contained.

Thanks for this suggestion – we have included and linked to a supplementary table outlining the key properties of existing approaches, borrowing heavily from a similar object in Diaz-Uriarte and Johnston, 2024.

A separate and expanded section or box, much closer to the beginning of the "Methods" section about the "visualization" tools is, IMHO, opinion necessary. Without it, interpreting the figures takes quite an effort.

This is a great suggestion, thanks. We have included a table giving the visualisation types, where each is used in the article, and details on their interpretation.

Most figures could be split in two, with more explanation in the captions, especially when the visualization requires much interpretation.

Following the above, we have now given each figure type a specific name and refer in each caption to the suggested table where visualisations are described and explained, and added detail to the captions to ensure that all styling aspects are explained (sorry for missing a few details previously!). We have kept the figure structure intact – while we appreciate that several figures are rather busy, we think the current allocation of "figure" to "content" is appropriate for the rhetoric of the manuscript and that splitting figures dealing with the same content might change this balance of emphasis for the reader.

Finally, I would suggest to make the tool available on BioConductor; it is already an R application after all.

Good suggestion, thanks -- we're working on adapting the code to the specific criteria of BioConductor and hope that the process of being included in the development branch will be underway during this round of review.

Reviewer #2: The authors proposed an extension of HyperTraPS (Hypercubic Transition Path Sampling) to the case of continuous time dynamics. The original HyperTraPS method is a Bayesian inference framework which models the accumulation of features in disease progression in evolutionary biology with a discrete time Markov chan, where the space of states is visualised as an hypercube in which vertices are the accessible states of the system and edges the transition steps between them. In their extension to continuous the processes, the authors provide a semi-analytical approach to compute

the transition probabilities between the states of the hypercube. The transition between states are assumed to be Poissonian and the waiting time distribution of a single trajectory ais modelled as an hyperexponential distribution, while the marginalization integral summing over all possible trajectories connecting different states are approximated by path sampling on the hypercube. Given the tipically large number of states, in order to reduce the space of allowed trajectories, the authors introduced what they called "compatibility condition", meaning that in a Markov chain step the system can acquire at most a feature in accumulation processes, or lose at most a feature in loss processes.
The authors proposed different type of parametrizations of the rates of the Markov chain which allow to capture higher order interactions between features and different regularization schemes as recursive feature elimination or additional terms in the likelihood penalizing model complexity during the learning process, which relies on a MCMC framework. The method is tested by the authors on simulated data increasing number of features and proposed two case of study in cancer progression of acute myeloid leukaemia and antimicrobial resistance genes acquisition in tubercolosis. The authors exploited these datasets to test the performance of the tools in predicting future and unobserved prediction in accumulation dynamics.
I appreciated the effort of the authors to extend HyperTraPS and establish a continuous time framework to study disease progression. In particular, I found very interesting the improvements regarding rates parametrization, the proposal of different regularisation schemes and the posterior estimates of disease pathways and feature correlations.

Thanks very much for your positive feedback and for your valuable and thought-provoking points below!

I have some questions regarding the use of the method in certain contexts that may be interesting in cancer evolution:

1) The concept of time is never clarified through the paper. The authors mentioned two single cell datasets for which they have ancestral relations between the genomic variants. Do they assign to any variant a pseudo-time related to its occurrence location in the cell tree? Do they assign to group of variants a time window of arrival identified by the times of barcode insertions? I think the authors may clarify which time scale they consider in their case of studies.

Thanks – we should indeed have been clearer here. In the tuberculosis case study the "time" ordinate is based on genetic distance; we have added some content to this existing description of this picture and a scalebar and description in the associated figure.

In the cancer data we do not assume an explicit timescale. Other approaches (notably TreeMHN for this dataset) invoke a picture of sampling time – assumed to be an exponentially distributed random variable, as explicit timing information is not present in the source data. While we could readily incorporate such timing information if available, here we use the flexibility of our time window approach and simply assume

that transitions occur at some time between an infinitely distant previous horizon and the moment of observation. We have added this detail.

In several of the synthetic studies exploring the performance of HyperTraPS-CT we use a precisely defined continuous timescale and explore the effect of assuming differing degrees of uncertainty on observations associated with this timescale (Supp Figs. 2, 5).

2) The authors assumed a compatibility condition in order to reduce the space of allowed trajectories of the system. This implies that the system can only acquire variants in accumulation processes, one per each step of the trajectories, and can never lose them. However, this may not be true, since evolution is always branched ([1]), and two populations collected at different time points will have a common ancestor and a set of common variants, the ones inherited by the common ancestor, and possibly a subset of private variants that appear only in one of the two samples. So, in general it is not true that the genomic state of system at the second time point has the genomic state at the first time point as boundary condition. Conversely, both the states should be considered as independent evolution of the system from a common boundary condition given by their common ancestor. Is it possible to account for such aspect of longitudinal data in the present framework?

Thanks for this important point. We do indeed assume that descendant observations reflect independent evolutionary lineages from their common ancestor. However, in case where ancestral states are not observed (e.g. our evolutionary case study), we are faced with the question of how to reconstruct these ancestral states. Here we indeed invoke a model with assumptions: effectively a maximum parsimony picture, where an ancestor is assumed to have a feature if and only if both its descendants have that feature. If only one descendant has the feature, that feature is assumed to be a "private variant" as the reviewer describes.

This model means that we will undercount cases of parallel/convergent evolution, where the same feature is acquired independent by the two descendant lineages. A more sophisticated (perhaps probabilistic) approach to ancestral state reconstruction would be more robust to this undercounting, but would also give rise to more putative transition sets to explore, increasing the complexity of the problem. A midway approach would be to (probably very wrongly) assume that every observation is independent, so that *all* positive observations of features reflect independent acquisitions (no inheritance). Then, inferences that are consistent across our current "no convergence" approach and this "completely convergent" approach could be reported as likely true regardless of the true generative dynamics. We have added this point to the discussion.

3) The authors implicitly assume that the population has no sub clonal structure and a system can be identified with a set of variants, which are therefore clonal. This may be not true in general [1] and it has been observed that a tumour may have polyclonal structure at detection time. In such scenario, the state of the system should be thought

as a mixture of states, with some variants present only in a subset of cells. Can the present framework account for polyclonal states of the cell population?

Broadly yes -- the framework can be applied immediately to systems that are a mixture of states. It is the interpretation of the mechanism and process that would then change, as it would not apply to (for example) within-cell gains; we would be using the model to describe the observational process as it happens on the mixture of states. That these processes are modelled using bulk samples is explicit in other cancer progression papers (e.g., Schill et al., 2024; Angaroni et al., 2021; Nicol et al., 2021). We've recently spent some time thinking about and surveying the consequences and limits for interpretation of this family of model for such heterogeneous data (Diaz-Uriarte and Johnston, 2024) – we've added a discussion point and a link to this literature throughout this comment and response.

Schill, R., Klever, M., Lösch Andreas, Hu, Y. L., Vocht, S., Rupp, K., Grasedyck, L., Spang, R., & Beerenwinkel, N. (2024). Overcoming observation bias for cancer progression modeling. In J. Ma (Ed.), Research in computational molecular biology (pp. 217–234). Springer Nature Switzerland. https://doi.org/10.1007/978-1-0716-3989-4_14

Angaroni, F., Chen, K., Damiani, C., Caravagna, G., Graudenzi, A., & Ramazzotti, D. (2021). Pmce: Efficient inference of expressive models of cancer evolution with high prognostic power. Bioinformatics, 38(3), 754–762. https://doi.org/10.1093/bioinformatics/btab717

Nicol, P. B., Coombes, K. R., Deaver, C., Chkrebtii, O., Paul, S., Toland, A. E., & Asiaee, A. (2021). Oncogenetic network estimation with disjunctive bayesian networks. Computational and Systems Oncology, 1(2), e1027. https://doi.org/10.1002/cso2.1027

Diaz-Uriarte, R., & Johnston, I. G. (2024). A picture guide to cancer progression and monotonic accumulation models: Evolutionary assumptions, plausible interpretations, and alternative uses (arXiv:2312.06824). arXiv. https://doi.org/10.48550/arXiv.2312.06824

4) Is it possible from the estimated feature correlations and predicted pathways to distinguish between drivers and passenger variants? The former are intended as genomic alterations that drive the evolution, providing fitness advantage or resistance, while the latter are variants that do not confer any fitness advantage [1]. The passenger variants that arrive on the genome of a cell before this acquires a driver raise their frequency [3] and in case of clonal sweep are found to be clonal at sample collection, even if they do not have any role in driving the disease progression. I think it would very interesting if it was possible to predict from the Markov chain dynamics the role of the variants and in particular which ones have a driving roles in the different trajectories of the disease progression and which ones are simply passengers.

References

[1] Turajlic, S., Sottoriva, A., Graham, T. et al. Resolving genetic heterogeneity in cancer. Nat Rev Genet 20, 404–416 (2019). https://doi.org/10.1038/s41576-019-0114-6

[2] Williams, M.J., Werner, B., Heide, T. et al. Quantification of subclonal selection in cancer from bulk sequencing data. Nat Genet 50, 895–903 (2018). https://doi.org/10.1038/s41588-018-0128-6

A really interesting point – related to one we have recently considered and discussed in Diaz-Uriarte and Johnston, 2024. One (overly simple) answer is that in the characterised set of influences between features in the inference process, one would expect “passengers” to have few outgoing edges (no influences on other features) and “drivers” to have more outgoing edges (influencing several other features). Such features with strong effects could be candidates for drivers. **But** we could see strong effects for very different reasons, and there could be drivers that show no strong effects.

Suppose event A has only a moderate effect on growth rate (i.e., has a small positive fitness contribution) and event B is highly deleterious, but the combination of A and B has a very large growth rate (i.e., fitness of genotype AB is much larger than that of A). What HyperTraPS-CT will tell us is that A has a strong effect on the downstream acquisition of B. But A is not really a "driver" in the usual sense (i.e., using the reviewer's words, it is not "[a genomic alteration] that drive[s] the evolution, providing fitness advantage or resistance").

Suppose, in contrast, that C is a driver in the usual sense, and it leads to a very large increase in population size. To make the scenarios simple, further suppose that once C is gained, nothing is gained from any additional mutations in terms of increases in growth rate, so additional mutations are either deleterious or neutral. Now, after C is mutated, the population will grow in size. As the population is now much larger, over the whole population, neutral mutations will appear sooner (given a constant mutation rate, we have more cells to have that mutation appear), but none of these additional mutations confer any benefit and their fate follows that of neutral mutations. So C is not making any difference for acquiring other mutations beyond the generic effect of "the population is larger".

In the above scenario, consider now what happens with passengers. As the reviewer explains, these neutral mutations "that arrive on the genome of a cell before this acquires a driver raise their frequency". However, over multiple patients, we expect there to be no consistent pattern to these "hitchhiking" events, so we would not find that C makes any difference for acquiring specific neutral mutations nor that specific neutral mutations make any difference for acquiring C.

As a third scenario, consider D as a driver in the usual sense. E, on its own, is deleterious, but the combination of D and E provides a further fitness advantage. Now, we will see a large effect of D on E. Acquiring D leads, quickly, to further adding E.

Thus, the precise relation between driver and passengers and the rates estimated by these methods is not simple, and can depend strongly on the details of the evolutionary model. This is why it is best to think of these methods as modelling "difference making for acquiring other events". In terms of fitness, event X increases the probability of acquiring event Y if the XY genotype has larger fitness than X and much larger fitness than Y (Y on its own might even be deleterious).

We've tried to concisely discuss this point in the manuscript, mentioning the possible connection between inferred influences and driver/passenger status but cautioning against a direct interpretation – and linked to the manuscript where we unpack the discussion in rather more depth. We've also submitted an update to our parallel preprint where we make connections between accumulation models and fitness, based on this discussion.
